# Timing of Chemotherapy and Radiotherapy Following Breast-Conserving Surgery for Early-Stage Breast Cancer: A Retrospective Analysis

**DOI:** 10.3389/fonc.2020.571390

**Published:** 2020-09-23

**Authors:** Si-Ye Chen, Yu Tang, Shu-Lian Wang, Yong-Wen Song, Hui Fang, Jian-Yang Wang, Hao Jing, Jiang-Hu Zhang, Guang-Yi Sun, Xu-Ran Zhao, Jing Jin, Yue-Ping Liu, Bo Chen, Shu-Nan Qi, Ning Li, Yuan Tang, Ning-Ning Lu, Hua Ren, Zi-Hao Yu, Ye-Xiong Li

**Affiliations:** Department of Radiation Oncology, National Cancer Center/National Clinical Research Center for Cancer/Cancer Hospital, Chinese Academy of Medical Sciences and Peking Union Medical College, Beijing, China

**Keywords:** breast neoplasm, breast-conserving surgery, chemotherapy, radiotherapy, timing

## Abstract

**Purpose:**

To investigate the effect of chemotherapy and radiotherapy timing after breast conserving surgery (BCS) on recurrence and survival of women with early-stage breast cancer.

**Patients and Methods:**

We retrospectively analyzed 900 patients who underwent BCS followed by both adjuvant chemotherapy and radiotherapy. Of these, 488 women received chemotherapy first (CT-first group) while the other 412 received radiotherapy first (RT-first group). Locoregional recurrence (LRR), distant metastasis (DM), disease-free survival (DFS), and overall survival (OS) rates were calculated using the Kaplan-Meier method and further confirmed with propensity-score matching (PSM) and the Cox proportional hazards model. The optimal cut-off value of interval time from surgery to the start of chemotherapy was calculated by Maxstat.

**Results:**

The median follow-up was 7.1 years. In pre-match analysis, the CT-first group had a significantly higher 8-year DFS than the RT-first group (90.4% vs. 83.1%, *P* = 0.005). PSM analysis of 528 patients indicated that the 8-year DFS (91.0% vs. 83.3%, *P* = 0.005) and DM (8.6% vs. 14.6%, *P* = 0.017) were significantly better in the CT-first group, but that the OS (*P* = 0.096) and LRR (*P* = 0.434) were similar. We found the optimal cut-off value of interval from surgery to chemotherapy was 12 weeks. Patients starting chemotherapy later than 12 weeks after surgery had significantly inferior survival outcomes.

**Conclusion:**

For women with breast cancer who require both chemotherapy and radiotherapy after BCS, adjuvant chemotherapy should be started within 12 weeks. Delaying the initiation of radiotherapy, for administration of long-course chemotherapy, does not compromise outcomes.

## Introduction

In patients with early-stage invasive breast cancer, breast-conserving therapy offers a similar overall survival to mastectomy (1). Postoperative radiotherapy remains an integral part of breast-conserving therapy, providing remarkably consistent local control and overall survival (2–4). Besides radiotherapy, patients at high risk are always recommended chemotherapy due to the substantial reduction of the risk of relapse and death (5, 6). For those requiring both radiotherapy and chemotherapy after breast conserving surgery (BCS), the optimal sequence of adjuvant therapy needs to be investigated.

Early randomized trials comparing concurrent with sequential chemotherapy and radiotherapy after BCS found no significant difference in survival, but detrimental effects on long-term late toxicities in patients receiving concurrent treatment (7–10). Thus, adjuvant concurrent chemoradiotherapy is generally not recommended in women with breast cancer after BCS. As for sequential treatment, only one small randomized trial has yet evaluated the effect of sequencing radiotherapy and chemotherapy with an anthracycline-based regimen in breast cancer after BCS, and found no significant differences in the rates of freedom from any adverse event or death (11). In addition, retrospective studies demonstrated inconsistent findings owing to a heterogeneous population in terms of patient characteristics, chemotherapy regimens, and radiation techniques (12–17). Improvements in both chemotherapy and radiotherapy, such as taxane-based chemotherapy and hypofractionated radiotherapy, have changed early breast cancer treatment practice (16, 18). However, the optimal sequence of adjuvant treatment needs to be further investigated. Therefore, we conducted this study to determine the optimal timing of initiation of chemotherapy and radiotherapy after BCS.

## Patients and Methods

### Patient Selection

**FIGURE 1:**
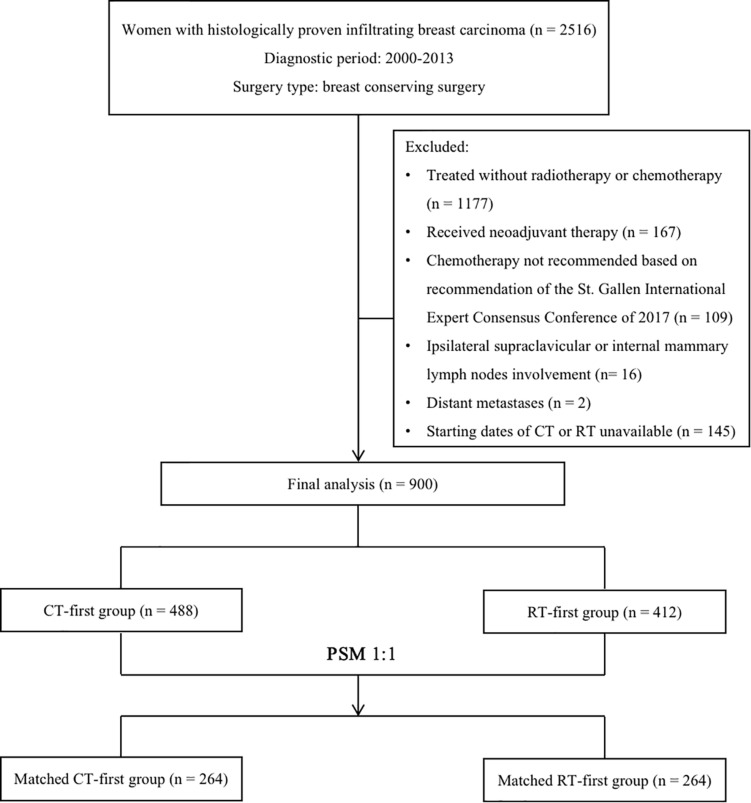
Trial profile.

We retrospectively reviewed women with histologically proven infiltrating breast carcinoma treated in the Cancer Hospital of Chinese Academy of Medical Sciences between January 2000 and December 2013. Altogether, 1339 women over 18 years old who received BCS followed by adjuvant chemotherapy and radiotherapy were identified by medical profiles. We excluded patients who received neoadjuvant therapy (*n* = 167), those whose chemotherapy should be withdrawn according to the latest recommendation of the St. Gallen International Expert Consensus Conference (*n* = 109) (19), those with ipsilateral supraclavicular or internal mammary lymph node involvement (*n* = 16), and those with distant metastases at initial diagnosis (*n* = 2). Patients were also excluded when the initiation date of chemotherapy or radiotherapy was unknown (*n* = 145). A final total of 900 eligible patients were included in this study. Among them, 488 women received adjuvant chemotherapy first (CT-first group) while the other 412 received radiotherapy first (RT-first group, Figure 1).

### Outcome Definition and Statistical Analysis

Interval time from surgery to chemotherapy (SCIT) was defined as the time from BCS to the start of chemotherapy. Interval time from surgery to radiotherapy (SRIT) was defined as the time from BCS to the start of radiotherapy.

The endpoints included disease-free survival (DFS), overall survival (OS), locoregional recurrence (LRR), and distant metastasis (DM). DFS was defined as the time from surgery to the first evidence of recurrence (locoregional or distant) or death from any cause. OS was defined as the time from surgery to death from any cause. LRR was defined as any tumor recurrence within the ipsilateral breast, or within the axillary, supraclavicular, or internal mammary nodes during follow-up. DM was defined as any failure outside the locoregional area defined above.

Baseline clinical characteristics were compared between different groups using the Chi-square test. Survival curves were estimated by the Kaplan-Meier method and compared with a log-rank test. Cox proportional hazards regression was performed for multivariate analysis. To address the imbalance of potential confounders of pretreatment variables, one-to-one patient matching without replacement was performed to pair cohorts of each group with a caliper size of 0.001 by propensity-score matching (PSM). We also used the Maxstat method to identify the optimal cut-off value of SCIT for outcomes (17). Statistical analyses were performed using SPSS Statistics v25.0 (IBM Corp., Armonk, NY, United States) and the “Maxstat” and “Matching” packages in R v3.4.4^1^. A *p*-value < 0.05 was considered stastistically significant.

## Results

### Baseline Characteristics and Outcomes

The baseline characteristics of the whole group is shown in Table 1. The median age was 44 years (range, 20-74). The majority of patients were treated between 2008 and 2013 (75.6%), had stage I/II disease (89.0%), and had hormone receptor–positive disease (74.2%). We determined molecular subtypes by tumor grade, estrogen and progesterone receptors (ER/PR), and human epidermal growth factor receptor 2 (HER2) status. In the 884 (98.2%) patients who had these data available, 210 (23.3%) were classified as Luminal A, 287 (31.8%) Luminal B1, 149 (16.6%) Luminal B2, 62 (6.9%) HER2 overexpression, and 176 (19.6%) were triple negative.

**TABLE 1:** Baseline characteristics of all patients stratified by receiving chemotherapy or radiotherapy first before and after matching.

	Before match				After match			
		
	All	CT-first group	RT-first group	P*	All	CT-first group	RT-first group	P*
	(*n* = 900)	(*n* = 488)	(*n* = 412)		(*n* = 528)	(*n* = 264)	(*n* = 264)	
							
Characteristic	No. (%)	No. (%)	No. (%)		No. (%)	No. (%)	No. (%)	
Treatment period				<0.001				0.912
2000-2007	220 (24.4)	62 (12.7)	158 (38.3)		100 (18.9)	49 (18.6)	51 (19.3)	
2008-2013	680 (75.6)	426 (87.3)	254 (61.7)		428 (81.1)	215 (81.4)	213 (80.7)	
Age (years)				0.611				1.000
<40	271 (30.1)	143 (29.3)	128 (31.1)		151 (28.6)	76 (28.8)	75 (28.4)	
≥40	629 (69.9)	345 (70.6)	284 (68.9)		377 (71.4)	188 (71.2)	289 (71.6)	
Pathological T stage				0.098				0.441
T1	635 (70.6)	335 (68.6)	300 (72.8)		377 (71.4)	184 (69.7)	193 (73.1)	
T2	265 (29.4)	153 (31.4)	112 (27.2)		151 (28.6)	80 (30.3)	71 (26.9)	
Pathological N stage				<0.001				0.695
N0	533 (59.2)	216 (44.3)	317 (77.0)		385 (72.9)	190 (72.0)	195 (73.9)	
N1-3	367 (40.8)	272 (55.7)	95 (23.0)		143 (27.1)	74 (28.0)	69 (26.1)	
Pathological staging				<0.001				1.000
I-II	801 (89.0)	400 (82.0)	401 (97.3)		505 (95.6)	251 (95.1)	254 (96.2)	
III	99 (11.0)	88 (18.0)	11 (2.7)		23 (4.4)	13 (4.9)	10 (3.8)	
Histological grade				0.265				0.789
1-2	549 (61.0)	306 (64.0)	243 (60.3)		324 (61.4)	160 (60.6)	164 (62.1)	
3	332 (36.9)	172 (36.0)	160 (39.7)		204 (38.6)	104 (39.4)	100 (37.9)	
Unknown	19 (2.1)	10 (2.0)	9 (2.2)		0	0	0	
Lymphovascular invasion				<0.001				1.000
Yes	66 (7.3)	51 (10.5)	15 (3.6)		23 (4.4)	11 (4.2)	12 (4.5)	
No	834 (92.7)	437 (89.5)	397 (96.4)		505 (97.1)	253 (95.8)	252 (95.5)	
Surgical margins				0.464				1.000
Positive	14 (1.6)	6 (1.2)	8 (1.9)		7 (1.3)	4 (1.5)	3 (1.1)	
Negative	886 (98.4)	482 (98.8)	404 (98.1)		521 (98.7)	260 (98.5)	261 (98.9)	
ER/PR status				0.321				1.000
Positive	668 (74.2)	369 (75.6)	299 (72.6)		376 (71.2)	188 (71.2)	188 (71.2)	
Negative	232 (25.8)	119 (24.4)	113 (27.4)		152 (28.8)	76 (28.8)	76 (28.8)	
HER2 status				0.664				0.271
Positive	211 (23.4)	119 (24.4)	92 (22.3)		134 (25.4)	73 (27.7)	61 (23.1)	
Negative	676 (75.2)	363 (74.4)	313 (76.0)		394 (74.6)	191 (72.3)	203 (76.9)	
Unknown	13 (1.4)	6 (1.2)	7 (1.7)		0	0	0	
Endocrine therapy				0.291				0.925
Yes	658 (73.1)	364 (74.6)	294 (71.4)		368 (69.7)	185 (70.1)	183 (69.3)	
No	242 (26.9)	124 (25.4)	118 (28.6)		160 (30.3)	79 (29.9)	81 (30.7)	
Anti-HER2-targeted therapy				0.540				0.259
Yes	110 (12.2)	63 (12.9)	47 (11.4)		74 (14.0)	42 (15.9)	32 (12.1)	
No	777 (86.4)	419 (85.9)	358 (86.9)		454 (86.0)	222 (84.1)	232 (87.9)	
Unknown	13 (1.4)	6 (1.2)	7 (1.7)		0	0	0	

CT, chemotherapy; RT, radiotherapy; ER, estrogen receptor; PR, progesterone receptor; HER2, human epidermal growth factor receptor 2. *Two-sided *P* values.

Patients received lumpectomy combined with either axillary dissection (*n* = 667, 74.1%) or sentinel lymph node biopsy (*n* = 233, 25.9%). All patients received adjuvant chemotherapy with a median of 6 cycles (range, 1-8). The most commonly used regimen was anthracycline combined with taxanes in 425 (47.2%) patients, followed by an anthracycline-based regimen in 253 (28.1%) patients, a taxane-based regimen in 171 (19.0%) patients, and other regimens in 51 (5.7%) patients. Nearly all patients with ER/PR positive tumor (*n* = 658/668, 98.5%) received endocrine therapy. While only half of patients with HER2-positive disease (*n* = 110/211, 52.1%) received anti-HER2-targeted therapy combined with chemotherapy, with the regimens of doxorubicin plus cyclophosphamide followed by paclitaxel plus trastuzumab (*n* = 93); or docetaxel, cyclophosphamide, and trastuzumab (*n* = 17).

All patients underwent whole breast irradiation with a boost to the tumor bed. Of them, 719 (79.9%) received tangential field-based 3D conformal or intensity-modulated radiation therapy, while the other 181 (21.1%) received 2D tangential field therapy only. Altogether, 756 (84.0%) patients received conventional fractionated radiation: 50 Gy in 25 fractions over 5 weeks for the whole breast plus a tumor bed boost of 10 to 20 Gy in 5 to 10 fractions. The remaining 144 (16.0%) received hypofractionated radiotherapy of 43.5 Gy in 15 fractions over 3 weeks for the whole breast, with a boost dose of 8.7 Gy in 3 fractions (18). Meanwhile, 123 (13.7%) patients, mostly with N2/3 disease, also received additional supraclavicular regional irradiation by conventional fractionation.

Within the median follow-up time of 7.1 years (range, 1.2-18.6), a total of 113 (12.6%) women relapsed. Of these, 18 (15.9%) were due to isolated LRR, 67 (59.3%) due to isolated DM, and 28 (24.8%) due to both LRR and DM. During follow-up, 70 (7.8%) patients died. Of these, sixty-seven were due to breast cancer, two were due to leukemia, and one was due to severe pneumonia. The OS and DFS rates were 97.0% and 91.7% respectively at 5 years, dropping to 92.1% and 86.5% respectively at 8 years. The LRR and DM rates were 3.0% and 7.1% at 5 years, increasing to 5.2% and 11.0% at 8 years.

### Treatment Intervals

**FIGURE 2:**
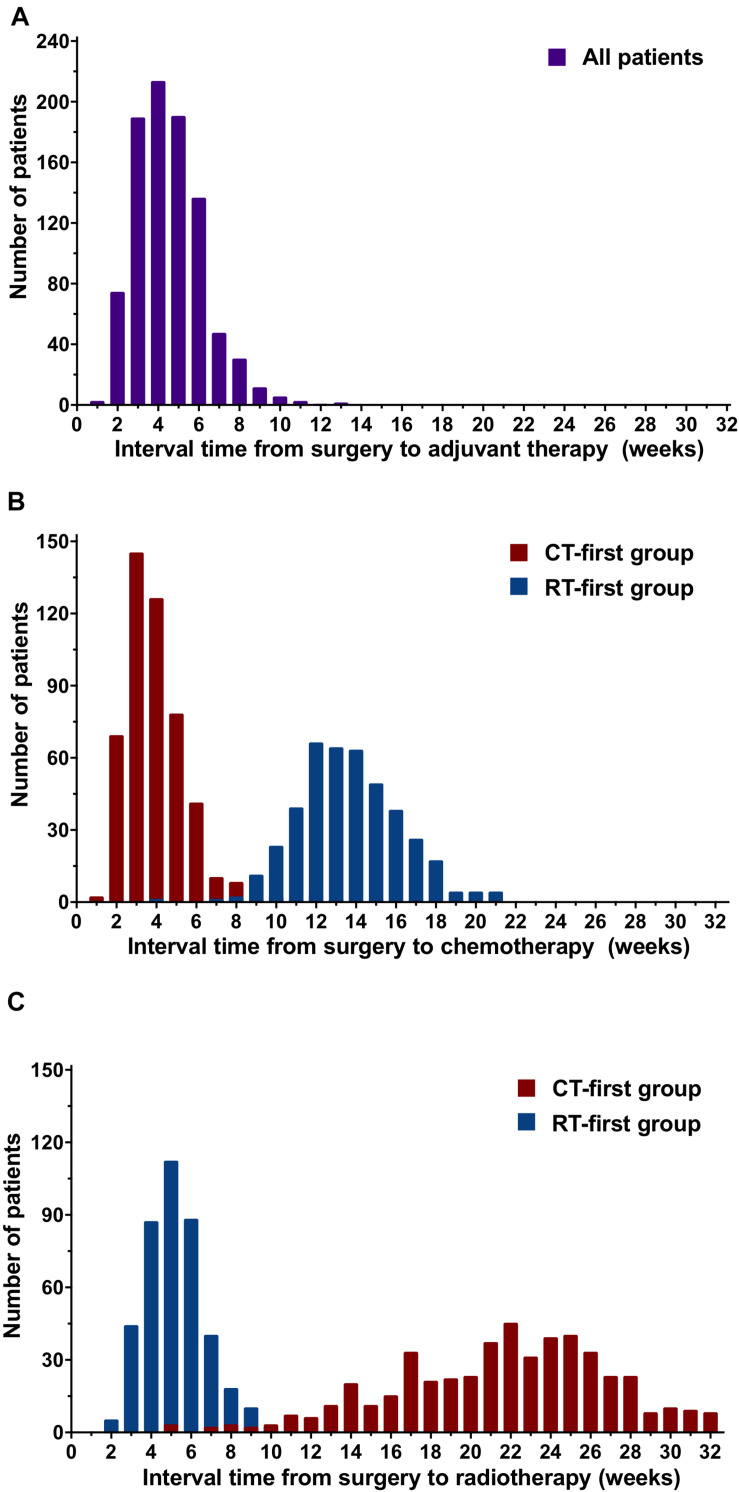
Distribution of the interval time from surgery to adjuvant treatment, chemotherapy, or radiotherapy for patients in the chemotherapy-first (CT-first) group and radiotherapy-first (RT-first) group. **(A)** Interval time from surgery to adjuvant treatment, **(B)** Interval time from surgery to chemotherapy (SCIT), **(C)** Interval time from surgery to radiotherap (SRIT).

The median interval between surgery and the start of adjuvant treatment was 4 weeks (range, 1-13; Figure 2A). The median interval time from surgery to chemotherapy (SCIT) was 6 weeks (range, 1-21) for all patients; for the CT-first group this was 4 weeks (range, 1-15); and for the RT-first group this was 13 weeks (range, 4-21; Figure 2B). The median interval time from surgery to radiotherapy (SRIT) was 14 weeks (range, 2-32) for all patients; for the CT-first group this was 22 weeks (range, 5-32); and for the RT-first group this was 5 weeks (range, 2-16; Figure 2C). All patients started radiotherapy within 32 weeks after BCS.

### Comparison of CT-First and RT-First Groups

**FIGURE 3:**
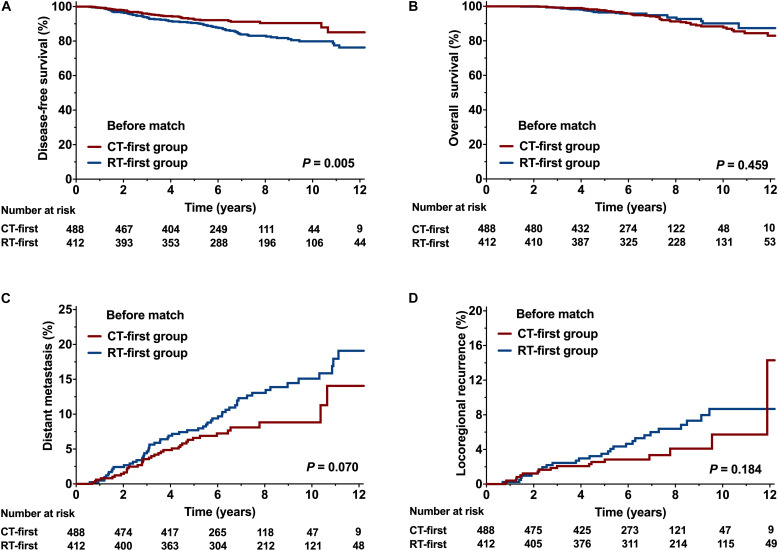
Comparison of the CT-first and RT-first groups before propensity score matching (PSM). **(A)** Disease-free survival (DFS), **(B)** Overall survival (OS), **(C)** Distant metastasis (DM), and **(D)** Locoregional recurrence (LRR).

Compared with the RT-first group, more patients in the CT-first group had high-risk factors, such as node-positive disease, stage III, and presence of lymphovascular invasion (Table 1). Since more patients in the CT-first group were treated in the time-period between 2008 and 2013 (Table 1), the median follow-up was 6.3 years (range, 1.2-15.8) for the CT-first group, whereas 8.5 years (range, 1.3-16.6) for the RT-first group. Patients in the CT-first group achieved a better DFS than those in the RT-first group (HR 0.58; 95% CI 0.39-0.85). The 5-year and 8-year DFS rates were 92.7% and 90.4% for the CT-first group, higher than those for the RT-first group (90.5% and 83.1% respectively, *P* = 0.005, Figure 3A). However, there was no significant difference between the two groups in terms of OS (*P* = 0.459, Figure 3B), DM (*P* = 0.070, Figure 3C), or LRR (*P* = 0.184, Figure 3D).

**FIGURE 4:**
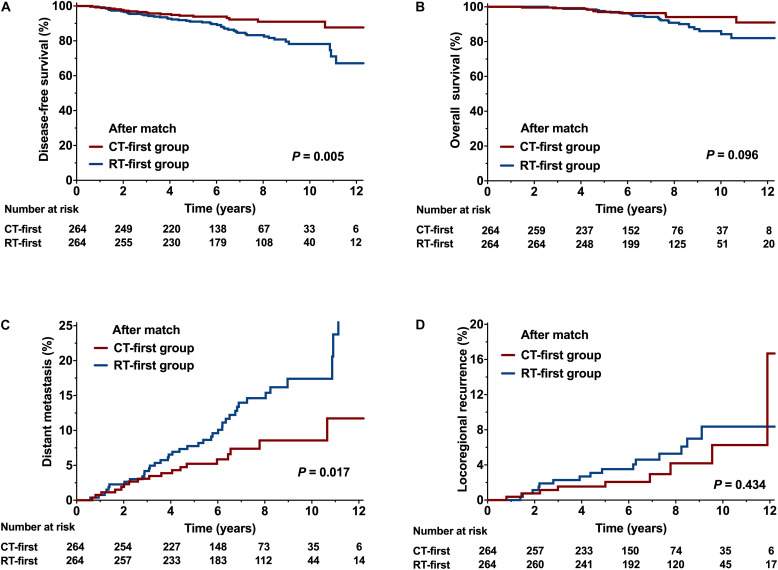
Comparison of the CT-first and RT-first groups after propensity score matching (PSM). **(A)** Disease-free survival (DFS), **(B)** Overall survival (OS), **(C)** Distant metastasis (DM), and **(D)** Locoregional recurrence (LRR).

Overall, 528 (58.7%) patients were selected by PSM, with 264 in each group. After adjusting for confounding variables, all clinical features were well balanced (Table 1). The CT-first group showed better DFS and DM compared with the RT-first group. The 8-year DFS rate of the CT-first group was 91.0%, significantly higher than the RT-first group (83.3%, *P* = 0.005, Figure 4A); the CT-first group had a 8-year DM rate of 8.6%, significantly lower than the RT-first group (14.6%, *P* = 0.017, Figure 4C). There was no significant difference in 8-year OS (94.2% and 90.9%, *P* = 0.096, Figure 4B) and 8-year LRR (4.2% vs. 5.3%, *P* = 0.434, Figure 4D) between the two groups.

### Optimal Interval Between Surgery and Chemotherapy

Maxstat indicated that the optimal cut-off value of SCIT influencing DFS was 12 weeks. Accordingly, we divided patients into two groups: SCIT < 12 weeks (*n* = 581) and SCIT ≥ 12 weeks (*n* = 319). Of the patients with a SCIT < 12 weeks, there were 484 (99.2%) in the CT-first group and 97 (23.5%) in the RT-first group. Compared with the group of SCIT ≥ 12 weeks, more patients in the group of SCIT < 12 weeks had high-risk factors (Supplemental Table).

**FIGURE 5:**
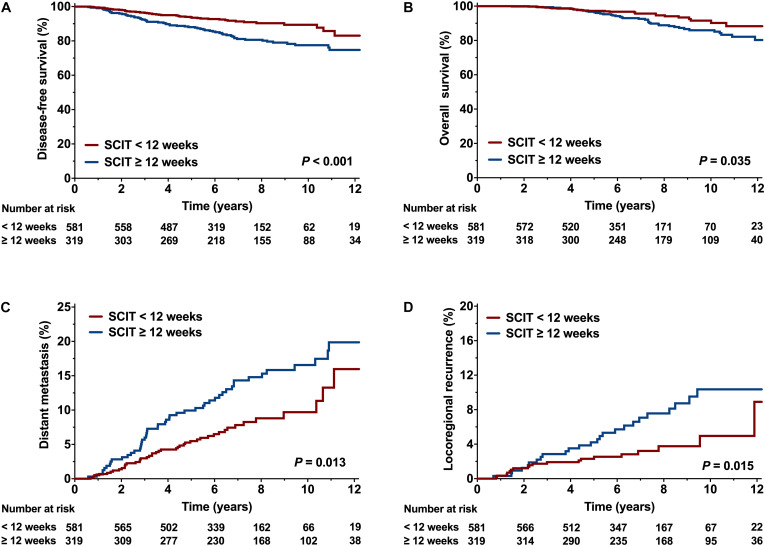
Comparison of survival curve between the interval time from surgery to chemotherapy (SCIT) < 12 weeks and SCIT ≥ 12 weeks. **(A)** Disease-free survival (DFS), **(B)** Overall survival (OS), **(C)** Distant metastasis (DM), and **(D)** Locoregional recurrence (LRR).

**FIGURE 6:**
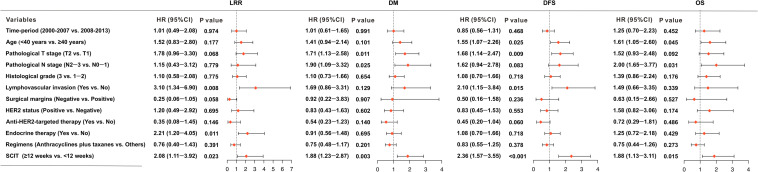
Forest plots indicating the independent prognostic effects of the interval time from surgery to chemotherapy (SCIT) < 12 weeks and SCIT ≥ 12 weeks, or clinical variables on locoregional recurrence (LRR), distant metastasis (DM), disease-free survival (DFS), and overall survival (OS).

Table 2 shows the results of univariate analysis of the association between clinical variables and survival outcomes. Compared to a SCIT < 12 weeks, a SCIT ≥ 12 weeks was associated with a significantly lower 8-year DFS rate (80.7% vs. 90.3%, *P* < 0.001; Figure 5A); and 8-year OS rate (88.6% vs. 94.7%, *P* = 0.035; Figure 5B); a higher 8-year DM rate (14.8% vs. 8.8%, *P* = 0.013; Figure 5C) and 8-year LRR rate (7.6% vs. 3.8%, *P* = 0.015; Figure 5D). Since 98.5% of hormone receptor-positive patients received endocrine therapy, the variable of hormone receptor was excluded in the multivariable analysis in order to avoid the interaction of these two variables. Multivariable analysis demonstrated that a SCIT ≥ 12 weeks was independently associated with increased risk of LRR (HR 2.08, 95% CI 1.11-3.91, *P* = 0.023) and DM (HR 1.89, 95% CI 1.23-2.87, *P* = 0.003), as well as adverse DFS (HR 2.35, 95% CI 1.57-3.55; *P* < 0.001) and OS (HR 1.88, 95% CI 1.13-3.11; *P* = 0.015; Figure 6).

**TABLE 2:** Univariate analysis of the association between clinical variables and survival outcomes for all patients.

	LRR		DM		DFS		OS	
							
Variables	HR (95% CI)	*P*	HR (95% CI)	*P*	HR (95% CI)	*P*	HR (95% CI)	*P*
**Treatment period**								
2008-2013 vs. 2000-2007	0.59 (0.31-1.11)	0.101	0.87 (0.55-1.37)	0.544	0.64 (0.43-0.96)	0.031	1.17 (0.67-2.03)	0.583
**Age (years)**								
≥ 40 vs. < 40	0.57 (0.32-1.02)	0.058	0.66 (0.44-1.00)	0.052	0.59 (0.41-0.86)	0.005	0.59 (0.36-0.94)	0.027
**Pathological T stage**								
T2 vs. T1	1.99 (1.11-3.58)	0.019	1.73 (1.15-2.61)	0.019	1.78 (1.23-2.60)	0.003	1.61 (1.02-2.60)	0.044
**Pathological N stage**								
N2-3 vs. N0-1	0.96 (0.38-2.43)	0.933	1.67 (1.05-2.88)	0.04	1.41 (0.84-2.36)	0.193	1.90 (1.05-3.30)	0.045
**Histological grade**								
3 vs. 1-2	1.35 (0.74-2.46)	0.334	1.13 (0.75-1.71)	0.551	1.07 (0.73-1.57)	0.717	1.42 (0.88-2.30)	0.169
**Lymphovascular invasion**								
No vs. Yes	0.41 (0.18- 0.92)	0.029	0.62 (0.32-1.19)	0.151	1.80 (1.01-3.22)	0.045	1.49 (0.68-3.25)	0.321
**Surgical margins**								
Negative vs. Positive	0.24 (0.06-1.00)	0.05	0.62 (0.15-2.52)	0.503	0.50 (0.16-1.58)	0.241	0.51 (0.12-2.06)	0.341
**ER/PR status**								
Negative vs. Positive	1.39 (0.75-2.58)	0.295	0.92 (0.57-1.47)	0.718	0.92 (0.59-1.41)	0.688	1.20 (0.72-2.00)	0.491
**HER2 status**								
Negative vs. Positive	1.32 (0.61-2.83)	0.484	1.41 (0.82-2.42)	0.211	1.53 (0.92-2.53)	0.101	1.17 (0.67-2.06)	0.582
**Endocrine therapy**								
No vs. Yes	1.90 (1.06-3.42)	0.032	0.90 (057-1.43)	0.898	1.04 (0.69-1.57)	0.865	1.18 (0.71-1.96)	0.528
**Anti-HER2-targeted therapy**								
No vs. Yes	2.72 (0.66-9.23)	0.168	1.90 (0.83-4.34)	0.13	2.29 (1.01-5.22)	0.049	1.44 (0.58-3.60)	0.431
**Chemotherapy regimens**								
Others* vs. Anthracyclines plus taxanes	1.09 (0.61-1.97)	0.774	0.87 (0.58-1.31)	0.497	1.04 (0.71-1.51)	0.855	0.83 (0.51-1.35)	0.449
**SCIT**								
≥ 12 weeks vs. < 12 weeks	2.06 (1.14-3.74)	0.017	1.67 (1.11-2.51)	0.014	2.01 (1.38-2.93)	< 0.001	1.68 (1.04-2.73)	0.037

LRR, locoregional recurrence; DM, distant metastasis; DFS, disease-free survival; OS, overall survival; HR, hazard ratio; CI, confidence interval; ER, estrogen receptor; PR, progesterone receptor; HER2, human epidermal growth factor receptor 2; SCIT, interval time from surgery to chemotherapy. *Other regimens include anthracycline-based regimen only, taxane-based regimen only, or other unknown regimens.

## Discussion

The optimal timing and sequence of chemotherapy and radiotherapy after BCS for early stage breast cancer has not been well defined, especially in the modern treatment era. This retrospective study demonstrated that patients receiving chemotherapy first had better DFS than those receiving radiotherapy first. Chemotherapy delayed beyond 12 weeks after BCS had a significant adverse effect on clinical outcomes.

Previous reports found conflicting results: some studies found that delaying the initiation of radiotherapy after BCS led to an increased risk of local failure (12, 20, 21), while others showed that radiotherapy started after the completion of chemotherapy but within 7 months after surgery did not affect tumor control or survival (14, 15, 22). Most patients in these studies were treated with outdated chemotherapy regimens (cyclophosphamide, methotrexate, fluorouracil; CMF); and endocrine therapy was not widely used. Recent studies using anthracycline-based regimens did not find an increased risk of local recurrence when radiotherapy was postponed after chemotherapy (11, 16, 23). Bellon et al. reported the findings of a prospective randomized trial on sequencing adjuvant treatment in node-positive patients after BCS, all of whom received 12 weeks of adjuvant anthracycline-based chemotherapy either before or after radiotherapy. There were no significant differences between the CT-first and RT-first arms in terms of time to any adverse event, distant metastasis, or death (11). The National Comprehensive Cancer Network (NCCN) breast cancer guidelines recommend that women receive adjuvant chemotherapy before radiotherapy after BCS based on the study by Bellon et al., but it does not have enough statistical power to rule out clinically important survival benefit for either sequence (11, 24). The addition of paclitaxel to anthracycline-based regimens was associated with better local control for node-positive disease (16). One study showed no effect on clinical outcomes of delaying radiotherapy for more than 32 weeks after BCS and completing four adjuvant cycles of doxorubicin/cyclophosphamide and four cycles of taxane (17). A study from the MD Anderson Cancer Center also showed no significant difference in recurrence-free survival between patients with node-negative disease who started radiotherapy < 25 weeks and ≥ 25 weeks after BCS when chemotherapy was delivered (25). In our study, the patients were young, 40.8% of them had node-positive disease, 11% had stage III disease, 36.9% had grade 3 tumors, and almost half received anthracycline plus taxane regimens. We also found that delaying the start of radiotherapy up to 32 weeks did not affect local tumor control.

Additionally, clarifying the pattern of recurrence is helpful for determining an adjuvant therapy schedule. Our study found that recurrence at a distant site was the main failure pattern for patients with early stage breast cancer, which is consistent with the literatures (26, 27), indicating the value of systemic therapy. Specifically, we found that delaying the initiation of chemotherapy beyond 12 weeks after BCS has an independently significant adverse effect on clinical endpoints after balancing these potential confounders by multivariable analysis. Therefore, early initiation of anthracycline and/or taxane-based chemotherapy regimens appears to be very important.

Consistent with our study, other research also showed that delaying the initiation of adjuvant chemotherapy had detrimental effects on survival outcomes. A systematic review indicated that overall survival (OS) decreased by 15% for every 4-week delay in initiation of chemotherapy with CMF or anthracycline-based regimens (28). Two large-cohort retrospective studies showed that initiation of adjuvant chemotherapy beyond 12 or 13 weeks from surgery was associated with inferior survival (29, 30). A pooled analysis of three clinical trials also demonstrated that SCIT longer than six weeks had a negative effect on OS and DFS in hormone receptor-negative patients, while delaying radiotherapy by more than six months after surgery did not affect outcomes in patients receiving long-course chemotherapy (31). All these studies included patients treated with both BCS and mastectomy, and the analysis did not differentiate between the two.

Almost all patients treated with BCS are recommended to receive radiotherapy, whereas in patients treated with mastectomy, radiotherapy is reserved for those at high risk. There is little dispute on the sequence of chemotherapy and radiotherapy in patients treated with mastectomy: chemotherapy followed by radiotherapy is commonly used in practice. Our study focused on patients treated with BCS, and emphasized the importance of early delivery of chemotherapy even in early-stage breast cancer. The important strengths of our study are: 1) the exclusion of patients who did not have an indication for chemotherapy based on the St. Gallen International Expert Consensus Conference of 2017 (19); and 2) most patients received anthracycline and/or taxane-based chemotherapy regimens; 3) the use of Maxstat to calculate the optimal cut-off value of SCIT. With hypofractionated radiotherapy becoming standard in breast cancer (18, 32, 33), the sequence of chemotherapy and radiotherapy will not be influenced to a great degree by the findings of our study, because patients could easily complete a 3-week radiotherapy course without delaying initiation of chemotherapy beyond 12 weeks after BCS.

Limitations of this study should be acknowledged. First, given the retrospective nature of the present study, selection biases cannot be avoided. CT-first group had more patients with high-risk features, and most patients in the CT-first group were treated during the recent time-period. However, after adjusting for the potential risk factors, including treatment period, we found SCIT of 12 weeks was an independent prognostic factor. Second, anti-HER2 targeted therapy has significantly improved DFS of patients with HER2-positive breast cancer in addition to chemotherapy (34). In our study, only 52.1% of patients with HER2-positive disease received anti-HER2 targeted therapy, which may exaggerate the effect of delay of chemotherapy due to the inadequate use of systemic treatment. Third, the 13-year span of patient inclusion was very long; therefore, changes in the diagnosis and treatment of breast cancer might have affected patients’ prognoses. Fortunately, we found that the treatment period did not significantly influence the prognoses of patients in this cohort. Last, as the majority of patients had stage I/II or ER/PR-positive diseases, who had a continuously risk of relapse beyond 5 years, thus longer-term follow-up is needed.

In summary, delaying the initiation of chemotherapy beyond 12 weeks after surgery is associated with inferior survival and tumor control in patients after BCS. However, delaying the initiation of radiotherapy up to 32 weeks, for administration of long-course chemotherapy, does not compromise patient outcomes. Longer-term follow up is warranted to validate our findings.

## Data Availability Statement

The raw data supporting the conclusions of this article will be made available by the authors, without undue reservation.

## Ethics Statement

The studies involving human participants were reviewed and approved by Ethics Committee of Cancer Institute and Hospital, Chinese Academy of Medical Sciences. Written informed consent for participation was not required for this study in accordance with the national legislation and the institutional requirements.

## Author Contributions

S-LW contributed and designed the research. S-LW and S-YC collected and analyzed data. S-YC, S-LW, YuT, and Y-XL wrote the article. All authors provided study materials or patients and approved the article.

## Conflict of Interest

The authors declare that the research was conducted in the absence of any commercial or financial relationships that could be construed as a potential conflict of interest.

## Footnotes

1 http://www.r-project.org/
