# Supplementary material for: Timing of Chemotherapy and Radiotherapy Following Breast-Conserving Surgery for Early-Stage Breast Cancer: A Retrospective Analysis
Source: Front Oncol. 2020 Sep 23;10:571390. doi: 10.3389/fonc.2020.571390 (PMC7538693; doi:10.3389/fonc.2020.571390)
Supplement: Supplementary file 1 [file Table_1.DOC]

**Supplemental table. Baseline characteristics of all patients stratified by SCIT<12 weeks and SCIT≥12 weeks.**

|  | **All**  **(n = 900)** |  | **SCIT<12 Weeks**  **(n = 581)** |  | **SCIT≥12 Weeks**  **(n = 319)** | ***P**** |
| --- | --- | --- | --- | --- | --- | --- |
| **Characteristic** | **No. (%)** |  | **No. (%)** |  | **No. (%)** |  |
| Treatment period |  |  |  |  |  | <0.001 |
| 2000-2007 | 220 (24.4) |  | 87 (15.0) |  | 133 (41.7) |  |
| 2008-2013 | 680 (75.6) |  | 494 (85.0) |  | 186 (58.3) |  |
| Age (years) |  |  |  |  |  | 0.255 |
| < 40 | 271 (30.1) |  | 167 (28.7) |  | 104 (32.6) |  |
| ≥ 40 | 629 (69.9) |  | 414 (71.3) |  | 215 (67.4) |  |
| Pathological T stage |  |  |  |  |  | 0.401 |
| T1 | 635 (70.6) |  | 404 (69.5) |  | 231 (72.4) |  |
| T2 | 265 (29.4) |  | 177 (30.5) |  | 88 (27.6) |  |
| Pathological N stage |  |  |  |  |  | <0.001 |
| N0 | 533 (59.2) |  | 287 (49.4) |  | 246 (77.1) |  |
| N1-3 | 367 (40.8) |  | 294 (50.6) |  | 73 (22.9) |  |
| Pathological staging |  |  |  |  |  | <0.001 |
| Ⅰ-Ⅱ | 801 (89.0) |  | 493 (84.9) |  | 308 (96.6) |  |
| Ⅲ | 99 (11.0) |  | 88 (15.1) |  | 11 (3.4) |  |
| Histological grade |  |  |  |  |  | 0.827 |
| 1-2 | 549 (61.0) |  | 357 (61.4) |  | 192 (60.2) |  |
| 3 | 332 (36.9) |  | 213 (36.7) |  | 119 (37.3) |  |
| Unknown | 19 (2.1) |  | 11 (1.9) |  | 8 (2.5) |  |
| Lymphovascular invasion |  |  |  |  |  | <0.001 |
| Yes | 66 (7.3) |  | 57 (9.8) |  | 9 (2.8) |  |
| No | 834 (92.7) |  | 523 (90.2) |  | 310 (97.2) |  |
| Surgical margins |  |  |  |  |  | 0.097 |
| Positive | 14 (1.6) |  | 6 (1.0) |  | 8 (2.5) |  |
| Negative | 886 (98.4) |  | 575 (99.0) |  | 311 (97.5) |  |
| ER/PR status |  |  |  |  |  | 1.000 |
| Positive | 668 (74.2) |  | 431 (74.2) |  | 237 (74.3) |  |
| Negative | 232 (25.8) |  | 150 (25.8) |  | 82 (25.7) |  |
| HER2 status |  |  |  |  |  | 0.459 |
| Positive | 211 (23.4) |  | 141 (24.3) |  | 70 (21.9) |  |
| Negative | 676 (75.2) |  | 432 (74.3) |  | 244 (76.5) |  |
| Unknown | 13 (1.4) |  | 8 (1.4) |  | 5 (1.6) |  |
| Endocrine therapy |  |  |  |  |  | 0.875 |
| Yes | 658 (73.1) |  | 426 (73.3) |  | 232 (72.7) |  |
| No | 242 (26.9) |  | 155 (26.7) |  | 87 (27.3) |  |
| Anti-HER2-targeted therapy |  |  |  |  |  | 0.241 |
| Yes | 110 (12.2) |  | 77 (13.3) |  | 33 (10.3) |  |
| No | 777 (86.4) |  | 497 (85.5) |  | 281 (88.1) |  |
| Unknown | 13 (1.4) |  | 7 (1.2) |  | 5 (1.6) |  |
| Chemotherapy regimens |  |  |  |  |  | <0.001 |
| Anthracyclines plus taxanes | 425 (47.2) |  | 330 (56.8) |  | 95 (29.8) |  |
| Others† | 475 (52.8) |  | 251 (43.2) |  | 224 (70.2) |  |

Abbreviations: SCIT, interval time from surgery to chemotherapy; ER, estrogen receptor; PR, progesterone receptor; HER2, human epidermal growth factor receptor 2.

*Two-sided P values.

†Other regimens include anthracycline-based regimen only, taxane-based regimen only, or other unknown regimens.
